# Spatially clustered patterns of suicide mortality rates in South Korea: a geographically weighted regression analysis

**DOI:** 10.1186/s12889-024-19899-4

**Published:** 2024-09-02

**Authors:** Eunah Kim, Seulgi Kim

**Affiliations:** 1 Present Address: Institute of Health and Environment, Seoul National University, 1, Gwanak-ro, Gwanak-gu, Seoul, 08826 Republic of Korea; 2 Institute of Health Policy and Management, Seoul National University Medical Research Center, 71 Ihwajang-gil, Jongno-gu, Seoul, 03087 Republic of Korea

**Keywords:** Suicide, Spatial analysis, Geographically weighted regression, Socioeconomic disparities in Health, Community Psychiatry

## Abstract

**Background:**

Suicide mortality remains a global health concern, and community characteristics affect regional variations in suicide. This study investigated spatially clustered patterns of suicide mortality rates in South Korea and evaluated the impact of community factors on suicide.

**Methods:**

Suicide mortality rates were estimated by sex, age group, and district, using the 2021 Cause of Death Statistics in South Korea from the MicroData Integrated Service. Community-determinant data for 2021 or the nearest year were collected from the Korean Statistical Information Service. The spatial autocorrelation of suicide by sex and age was examined based on Global Moran’s I index. Geographically weighted regression (GWR) was used to discern the influence of community determinants on suicide.

**Results:**

Suicide mortality rates were significantly higher among men (40.64 per 100,000) and adults over the age of 65 years (43.18 per 100,000). The male suicide mortality rates exhibited strong spatial dependence, as indicated by a high global Moran’s I with *p* < 0.001, highlighting the importance of conducting spatial analysis. In the GWR model calibration, a subset of the community’s age structure, single-person household composition, access to mental healthcare centers, and unmet medical needs were selected to explain male suicide mortality. These determinants disproportionately increased the risk of male suicide, varying by region. The GWR coefficients of each variable vary widely across 249 districts: aging index (Q1:0.06–Q3:0.46), single-person households (Q1:0.22–Q3:0.35), psychiatric clinics (Q1:-0.20–Q3:-0.01), and unmet medical needs (Q1:0.09–Q3:0.14).

**Conclusions:**

Community cultural and structural factors exacerbate regional disparities in suicide among men. The influencing factors exhibit differential effects and significance depending on the community, highlighting the need for efficient resource allocation for suicide. A regionally tailored approach is crucial for the effective control of the community’s mental health management system.

**Supplementary Information:**

The online version contains supplementary material available at 10.1186/s12889-024-19899-4.

## Introduction

Worldwide, there are over 700,000 deaths per year due to suicide [1], and these mortalities show distinct patterns according to age, sex, race/ethnicity, and region [2]. The COVID-19 pandemic also negatively affected mental health and suicide risk was rampant. The physical distancing policies disrupted social relations and resulted in social isolation, loneliness, and depression [3]. Suicide risk has increased both globally and locally, resulting in a growth in regional health inequality [4].

South Korea, recorded as having the highest suicide mortality among Organisation for Economic Co-operation and Development (OECD) countries, reached 24.6 suicide deaths per 100,000 in 2020, which is more than double the OECD average of 11.0 deaths per 100,000 [5]. Men have a higher risk of dying by suicide; in 2022, the male suicide rate (35.3 per 100,000) was more than twice that of women (15.1 per 100,000) [6]. The risk of suicide for both sexes tend to increase with age [7]. Additionally, South Korea is experiencing a continued rise in disease burden of suicide (years of life lost, YLL), which is projected to become the second-highest cause of YLL in 2040 [8]. The regional variation in YLL owing to suicide exceeded threefold across 237 districts in South Korea in 2016 [9].

Suicide mortality is influenced by contextual factors at the community or national level, as well as by individual factors. A recent systematic review of the social determinants of mental disorders presents a conceptual framework comprising demographic, economic, neighborhood, environmental events, and social and cultural domains [10]. Regarding macroeconomic indicators, the unemployment rate [11, 12], community deprivation levels [13], regional income levels [14], and the allocation of assistance for low-income groups [12] are correlated with suicidal behaviors and mortality. Neighborhood characteristics, including overcrowding in urban areas [15], the percentage of vacant houses, and access to parks [16], are associated with community suicide mortality rates. Access to local mental health and welfare facilities also influences community suicide rates [17]. Rural areas, susceptible to social deprivation [18] and with limited access to mental health services [19], are likely to experience increased suicide mortality. Cultural differences among ethnic minorities, immigrants, and indigenous peoples contribute to varying suicide rates, explaining global differences in suicide rates between Western and Eastern regions [20, 21].

From an ecological perspective, previous studies in Korea have attempted to address spatial concerns related to suicide mortality rates. Some researchers have focused primarily on spatially clustered patterns of suicide without sufficient consideration of the community factors that contribute to these tendencies [22, 23]. Other researchers have examined community determinants of suicide [14, 17, 24, 25], but their use of global coefficients in regression models assumed that the effects of these determinants were uniform across all regions. A recent study estimated the spatiotemporal association between suicide and social environments using the Laplace approximation by stratifying the dataset into age- and sex-specific groups as well as urbanization levels. [16]. However, these studies have been limited in accounting for spatial non-stationarity and geographical proximity between regions, and in illustrating local maps that might offer empirical and policy insights.

The severity or impact of suicide risk factors varies by community [26]. Geographically weighted regression (GWR) allows for local parameter estimates at each location, varying across areas, rather than applying a single global coefficient [27]. GWR is designed to answer the question, “Do relationships vary across space?” and examines spatial non-stationarity in the relationships between dependent and independent variables [28]. This local spatial statistical technique, in contrast to the global ordinary least squares model, can distinguish specific locations of interest and provide valuable insights for region-specific strategies. Visualizing GWR results enhances interpretation by incorporating spatial context and the unique characteristics of the study region [29].

This study aims to investigate vulnerable populations and locations related to suicide mortality and the varying effects of community factors on suicide in South Korea. We specify sensitive segments by sex and age based on spatially clustered patterns of suicide mortality. Subsequently, we identify community factors that explain the spatial non-stationarity between suicides and determinants. Finally, we examine local variations in how these community factors exert their effects across areas. These findings may enhance the understanding of efficient resource allocation or suicide prevention strategies tailored to specific regions.

## Methods

### Study area and population

As an ecological study, the dataset was aligned with the Korean administrative units, which comprised 17 cities and provinces and was further subdivided into 250 districts. We set these 250 districts as the units of analysis. When the data were reported for 229 districts or 255 community levels, these were duplicated or aggregated to obtain values per each district (as detailed in Appendix A).

This study covers the overall population (*N* = 51,333,252) in Korea for 2021, divided by sex (assigned sex at birth) and age group (15–34 years; 35–64 years; and 65 years and over). The study’s target population for applying GWR is determined by the results of spatial autocorrelation analysis, following these selection criteria:﻿ 1) the presence of spatial autocorrelation in the outcome variable; and 2) high prevalence rates of the outcome itself, highlighting the high-risk group to be studied.

### Suicide mortality

Suicide mortality data were obtained from the Cause of Death Statistics for 2021 in South Korea, which is annually reported by Statistics Korea based on death notifications. After submitting a designated form to the Micro Data Integrated System (MDIS; https://mdis.kostat.go.kr) for approval, cause of death data are provided by each code, sex, age group, and district as requested. The total number of deaths was 317,680 in a population of 51,333,252. Suicide was coded as intentional self-harm (X60–X84), and there were 13,352 deaths due to suicide. We stratified suicide mortalities by sex and age group, and estimated mortality rates (suicide death cases per 100,000 people) in each stratified group and district. Correlation between each group’s suicide mortality were investigated as presented in Appendix B.

### Community determinants

Regarding the ecological impacts on mental health described in a previous study [10], we identified five domains and collected eleven representative variables: demographic characteristics of the community (aging index, single-person household rate), economic characteristics (gross regional domestic product [GRDP], unemployment rate), neighborhood characteristics including infrastructure and availability of essential services (urbanization rate, road network index, number of psychiatric clinics and hospitals, unmet medical needs), environmental events (disruption of daily life due to COVID-19), and social and cultural characteristics (social trust, usage of psychiatric counseling). Data were sourced from the Community Health Survey (CHS; https://chs.kdca.go.kr) and a government open data portal, the Korea Statistical Information Service (KOSIS; https://kosis.kr). Detailed information on the data is provided in Appendix A.

After conducting the model specification procedure (as detailed in Appendix C) to identify the explanatory variable subset for GWR [30], a subset of four out of eleven variables was selected based on the minimum Akaike information criterion with correction (AICc)—1) aging index (ratio of + 65/<15 years old); 2) single-person households (percentage of the number of single-person households out of total types of households); 3) psychiatric clinics (number of psychiatric clinics and hospitals per 100,000 people); and 4) unmet medical needs (percentage of survey respondents who answered that they needed medical care in the past year, but did not receive it).

### Spatial autocorrelation

To measure the spatial autocorrelation of suicide mortality across 250 districts, we calculated the global Moran’s I by sex and age and derived a local Moran’s I (i.e., a local indicator of spatial association; LISA) cluster map. The global Moran’s I assesses the overall degree of spatial autocorrelation in the data. When this index shows non-significance, it implies that the suicide rates are randomly distributed across districts, not undergoing spatial processes. The LISA cluster map illustrates the presence and location of hot (or cold) spots where positive (or negative) values gather within adjacent regions [31]. As the Korean districts are irregular in shape and size, the spatial weight matrix was constructed using the first-order queen’s contiguity method [32], which defines neighbors as regions that share common boundaries and nodes.

### GWR model selection

GWR uses a moving-window weighting technique that localizes each model to the target locations. Because GWR uses relatively small samples (i.e., a local subset of the data) for each local parameter estimation, we conducted three procedures to enhance model performance and minimize the risk of local collinearity [28, 30, 33]. First, we conducted the GWR model selection procedure using the R function *model.selection.gwr* [30] to identify the optimal independent variable set based on the minimum AICc, as detailed in Appendix C. Second, various kernel functions can be utilized for spatial weighting, where the bandwidth controls the window size. We fitted each combination of adaptive or fixed bandwidth and bi-square or Gaussian kernel functions to the selected variable set, demonstrating relevant options based on goodness-of-fit statistics. Finally, observations with extreme values of GWR local R-squared were treated as outliers and removed, as described in Appendix D.

Consequently, the final GWR model in this study employed four explanatory variables as noted above using a fixed bandwidth and a Gaussian kernel function, and was fitted for 249 districts after removing one outlier. Sensitivity analysis using total variable set also supported the validity of the final model with four explanatory variables (presented in Appendix E). For a meaningful interpretation of results, we provide the results of GWR parameter estimates by mapping them with statistical significance as indicated by the local t-value [34].

All analyses were performed using SAS version 9.4, R version 4.3.1, and GeoDa version 1.22 software.

## Results

### Spatial autocorrelation of suicide mortality rates by sex and age

Table 1 presents the average suicide mortality rates by sex and age group at the district level and the spatial autocorrelation across 250 districts in 2021 in South Korea. There were 28.84 suicide mortalities per 100,000 people overall. Regarding the crude rates by sex and age groups, suicide mortalities were considerably higher in men (40.64 per 100 K) and adults over 65 years (43.18 per 100 K).

The study target was identified based on high spatial autocorrelation and prevalence rates, which are prone to spatial clustering. The spatial autocorrelation results (high global Moran’s I with *p* < 0.05) indicated potential study targets, including 1) men, 2) ages 15–34, 3) ages 35–64, and 4) the entire population. The 15–34 age group was not considered due to its relatively low suicide rates. Given the high correlation between the 35–64 age group and men, as well as between the overall population and men (as detailed in Appendix B), we assume that analyzing male suicides will closely mirror the suicide patterns in these segments. Therefore, we selected men as the target population for spatial analysis.

### Location of hot and cold spots of male suicide mortality

**Fig. 1:**
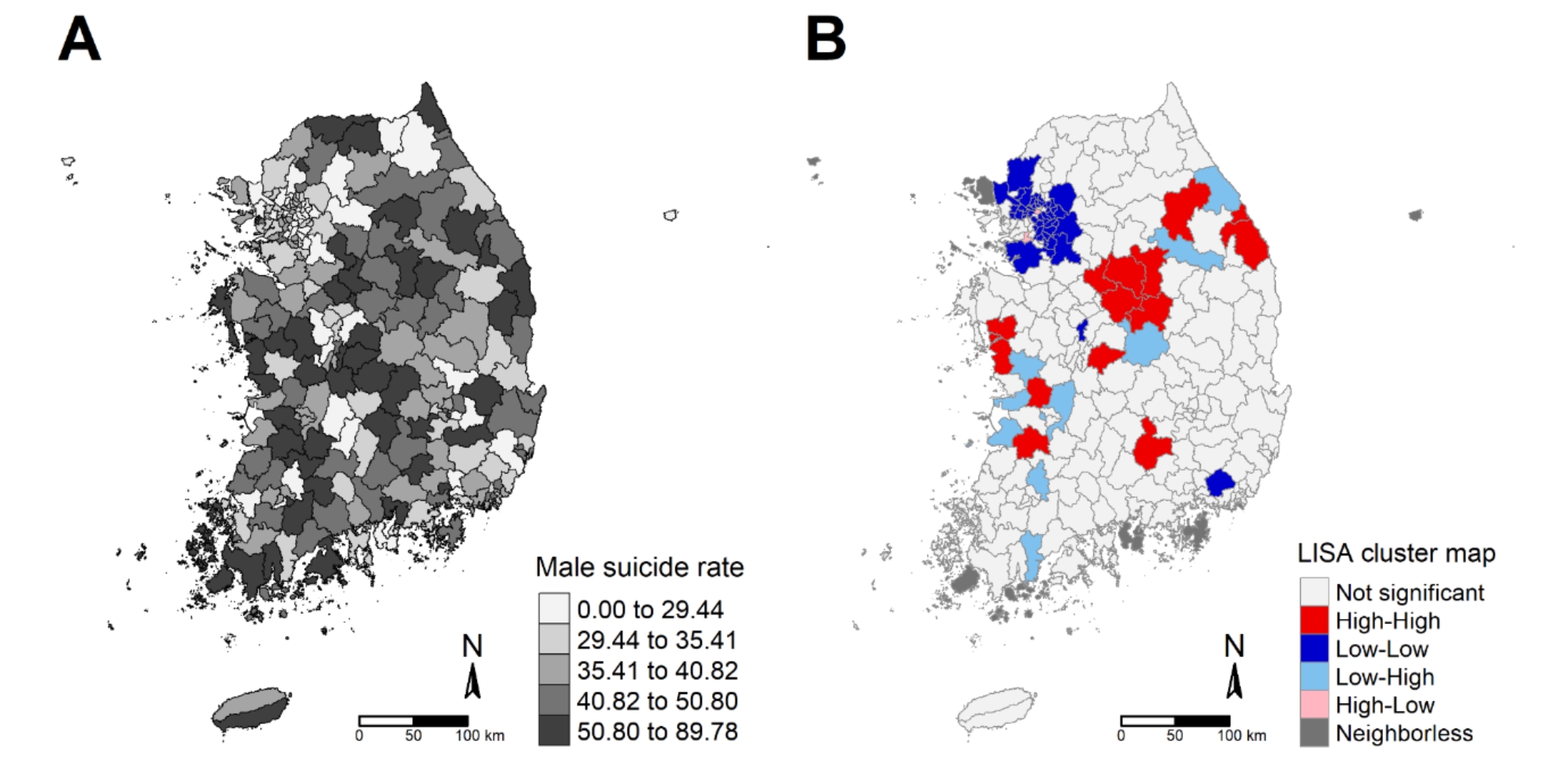
Geographical distribution of male suicide mortality rates (**A**) and its local Moran’s I cluster map (**B**) in South Korea (2021). **Notes**: Male suicide mortality rates in Fig. 1(A) were divided into quintiles (Q5) across 250 districts. Local Moran’s I in Fig. 1(B) was calculated based on the spatial weight of queen contiguity, and the significance of the index was tested using 999 permutation operations

**Fig. 2:**
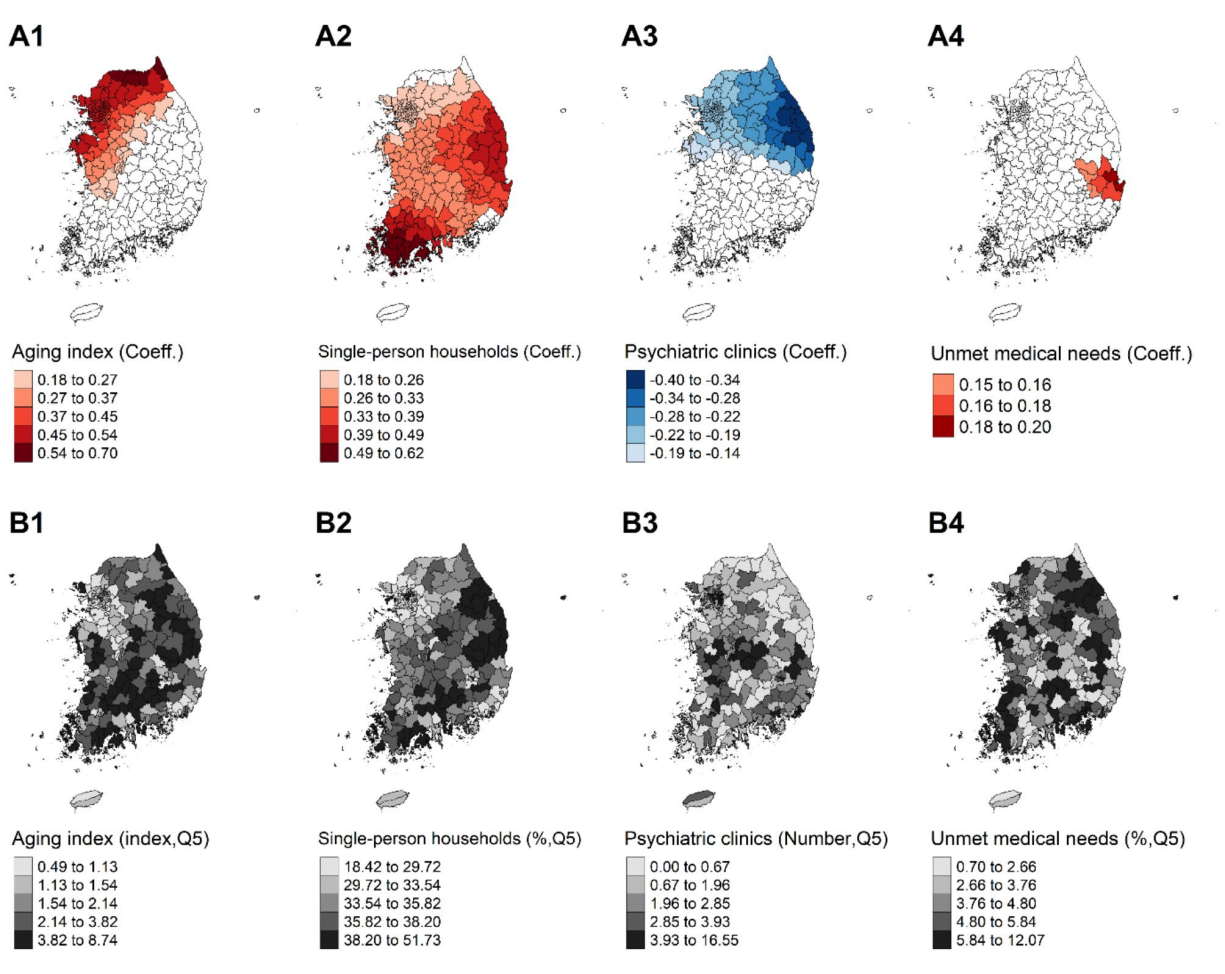
Spatial distributions of geographically weighted regression (GWR) coefficients (**A**) and crude rate (**B**) of each determinant of male suicide mortality rates in South Korea (2021). **Notes**: Character A indicates the spatially varying effect (GWR coefficient) of the determinant, only denoting the coefficients with statistical significance (local t-value > |1.96|) which were divided into classes using Jenks natural breaks classification method. B indicates the amount of distributed factor across regions, which were divided into quintiles. Each determinant is designated as a number: aging index, 1; percentage of single-person households (%), 2; psychiatric clinics and hospitals per 100,000 people, 3; unmet medical needs (%), 4

Figure 1 shows the geographical distribution and LISA cluster map of male suicide mortality. A detailed map of administrative boundaries is presented in Appendix F. There are “cold spots (spatial clusters of low values)” around the capital (Seoul) and metropolitan areas (Gyeonggi-do) of South Korea. By contrast, “hot spots (spatial clusters of high values)” were concentrated in the central, eastern, and coastal areas, which are relatively rural. Regarding the community characteristics described in Fig. 2B, the hot spot regions comprised older adults and had many single-person households, a lack of psychiatric clinics, and a high prevalence of unmet medical needs. Conversely, the cold spot regions were characterized by a younger population and abundant mental healthcare resources.

### Spatially varying relationships between community determinants and male suicide mortality

Table 2 presents the GWR results of community factors on male suicides. Figure 2A illustrates the geographical distribution of significant GWR coefficients, where local t-values exceed + 1.96 or fall below − 1.96. As a community’s population structure becomes younger, the risk of male suicide mortality decreases. GWR coefficients of the aging index were significant in 122 northern districts (near the capital of South Korea) and varied considerably from 0.058 (Q1) to 0.464 (Q3) across 249 districts (Fig. 2(A1)). Single-person households in the community increased the risk of male suicide mortality across 87.1% of the districts, with high coefficients in the southern areas called Jellanam-do (Fig. 2(A2)). The low number of psychiatric clinics was statistically associated with increased male suicide mortality, particularly in 122 out of 249 districts (49.0%), with varying GWR coefficients (Q1 to Q3: -0.201 to -0.014) across districts. The northeastern and coastal regions, specifically Gangwon-do, showed a strong correlation between the number of clinics and male suicide rates (Fig. 2(A3)), while other areas had coefficients relatively close to zero. Unmet medical needs were positively associated with male suicide rates, but the number of regions with statistical significance was only nine, centered in the Gyeongsangbuk-do and Daegu (Fig. 2(A4)). A local map with detailed information on GWR coefficients is provided in Appendix G.

## Discussion

We investigated the spatial patterns of suicide mortality and found spatial dependence on male suicides. The male mortality rate in 2021 in South Korea was approximately 2.4 times higher than that of women, and community determinants disproportionately increased the risk of male suicide based on region. These findings imply the spatial non-stationarity of the determinants’ effects: communities with prevalent single-person households were more vulnerable to male suicide, while younger populations near the capital were correlated with lower male suicide rates. The lower number of psychiatric clinics was highly associated with male suicide rates, particularly in the Gangwon-do region, while unmet medical needs were significantly associated, particularly near Daegu and Gyeongsangbuk-do region. These findings affirm the regionally dependent traits of male suicide, highlighting the need for countermeasures tailored to the community.

Understanding the differences between the two sexes in terms of suicide patterns and determinants is crucial for effective intervention strategies. In this study, spatial autocorrelation analysis indicated that male suicide rates, but not female suicide rates, had a geographically clustered pattern across regions in South Korea. Consistent with previous research, female suicides show less geographic concentration compared to male suicides [35–37]. At subregional levels, the number of female suicide deaths is significantly lower than that of male suicide deaths, resulting in a lack of detectable clusters [35]. Additionally, male suicides are more prominently affected by contextual determinants, including macro-societal and economic factors, than female suicides [38–41]. Some studies have explained men’s vulnerability to suicide within the cultural context, where the traditional hegemony of masculinity (gender role expectations) increases maladaptive coping strategies, such as alcohol abuse, in stressful situations and reluctance to seek help, consequently raising the suicide risk in men [12, 42, 43]. Even though there is no regional concentration of women’s suicide, given that the sex differences in suicidal progress pathways and effect factor mechanisms [44], further research on female suicidal behavior is necessary.

Of the community determinants collected for this study, four variables were fitted to the GWR model to explain male suicide. First, the aging index of the community was positively correlated with male suicide, suggesting a possible protective effect of a younger population. Notably, these significant associations (i.e., significant GWR coefficients) were concentrated near the capital and metropolitan cities. This can be supported by social explanations such as urban vitality’s impact on mental health, which are closely correlated with factors like the proportion of a younger population, population inflow, social infrastructure, and a complex interplay of economic and cultural phenomena [45]. Indeed, communities with high social vitality and abundant resources—such as social relationships [46], economic/commercial activity [47, 48], and accessibility to essential services [49]—affect residents’ mental health, including depressive symptoms, stress levels, and overall well-being. In particular, these community environments were strongly associated with men’s mental health [47, 50]. These findings emphasize the significant impact of urban environments on male suicide mortality.

Second, the proportion of single-person households in a community was associated with higher male suicide rates at the community level. These association were valid throughout the country but were particularly strong near the southwestern areas of South Korea, where single-person households were prevalent. Näher et al. [48] measured social isolation as the proportion of single-person household in a district and its district compositional effects were associated with increase in suicide rates [48]. Similarly, residing in cities with a higher proportion of multi-person households is linked to a reduced suicide risk, as it promotes greater social integration by enhancing community stability and cohesion and mitigating nonnormative and problematic behaviors [51]. Improving social connectivity and civic participation can reduce suicide across the rural–urban continuum [18]. As the number of single-person households has increased worldwide [52], suicide prevention strategies should take social isolation into account by targeting high-risk households.

Third, we observed region-specific associations between mental healthcare resources and male suicide mortality rates. The lower number of psychiatric clinics per residents was significantly correlated with an increased risk of male suicide, particularly in Gangwon-do region (northeast areas of South Korea). Given that these areas are of persistent concern as medically vulnerable areas in Korea [53], our results emphasize the need for timely access to mental healthcare to prevent suicide [19, 54]. In US counties, areas with mental health workforce shortages are associated with an increased rate of youth suicide [55]. Similarly, in Japan, regions with a higher ratio of psychiatrists to residents have significantly decreased suicide rates, underscoring the crucial role of mental health resource allocation [56]. For men in South Korea, receiving a psychiatric assessment from a psychiatrist reduces the risk of suicide among those who have deliberately harmed themselves before [57]. In a series of suicide processes—from feelings of sadness and depression to suicidal thoughts, suicide attempts, and mortality—contact with mental healthcare professionals is a crucial intervention that protects against the progress of suicidal behaviors [58]. This study supports the idea that geographically accessible and available mental health care facilities are fundamental for establishing community intervention strategies. It is important to increase the number of psychiatric clinics to ensure that the service is accessible to those in need.

Finally, the results show that unmet medical needs in South Korea were randomly distributed across regions. However, the significant associations (GWR coefficients) with male suicide were confined to the regions of south-east Korea, particularly Daegu and Gyeongsangbuk-do. Interestingly, these regions were the origins of the COVID-19 outbreak in 2020 in South Korea, and as COVID-19-related casualties surged in tertiary and nursing hospitals, strict control measures were implemented [59]. There was a substantial decline in primary care contacts, resulting in indirect death during the pandemic. The most significant reductions were observed in consultations for mental distress, including depression, self-harm, anxiety, and severe mental illness [60]. Our results showing a valid association between unmet medical needs and male suicide suggest that there might be indirect deaths attributed to suicides in 2021, particularly in regions severely affected by the pandemic.

This study highlights the regionality of male suicides, determined by a community’s cultural and structural factors. We considered spatial heterogeneity to explain male suicide patterns, and asserted that community factors influence men’s vulnerability to suicide. Two distinct aspects of GWR results highlight the importance of resource allocation policies [61]: the abundance of resources within the community, and the effectiveness of the resources in that region. If resources are ineffective in decreasing the suicide rate for a designated community, increasing the volume of resources in that region cannot prevent suicide. Therefore, healthcare resource policies should aim for efficient resource allocation, while considering the magnitude of the effect of the influencing factors tailored to specific regions.

This study has some limitations that require consideration when interpreting the results. We included four explanatory variables in the GWR model after conducting a forward stepwise model selection procedure. Due to the limited dataset of 250 observations and high correlations among the 11 variables, we selected the GWR model based on the AICc. However, the omitted variables could also provide valuable insights, indicating the need for further research. Additionally, due to data sharing policies related to suicides in this study, it was not possible to obtain suicide cases per district as a cross table by sex and age. By accumulating data over a long-term period, it would be possible to obtain and estimate more detailed segments’ standardized mortality rates. Finally, the results were limited to South Korea, restricting their generalizability globally. Future research in culturally diverse settings is needed to validate these findings.

## Conclusions

Community attributes influence suicide within a specific spatial range. The findings of this study demonstrate the presence of spatial clusters and regional inequalities in male suicides in South Korea, partially resulting from community characteristics. Therefore, efforts should be made to identify vulnerable rural areas with weak social networks and to develop resource allocation strategies for vulnerable males to ensure access to psychiatric clinics and meet their medical needs. We emphasize the importance of suicide prevention programs tailored to the community context.

**Table 1:** Average and spatial autocorrelation of suicide mortality by sex and age in 250 districts across South Korea (2021)

	Average suicide mortality rate (deaths per 100,000)		Spatial autocorrelation of suicide mortality		
	Mean	(S.D.)	Global Moran’s I	Z-score	*P*-value
**Overall**					
South Korea	28.84	(7.94)	0.231	5.696	< 0.001
**By sex**					
Male	40.64	(13.46)	0.211	5.230	< 0.001
Female	16.96	(6.68)	0.014	0.452	0.324
**By age (years)**					
Over 15–under 34 under	22.99	(12.43)	0.103	2.583	0.007
Over 35–under 64	31.18	(10.03)	0.182	4.497	< 0.001
Over 65	43.18	(15.52)	-0.002	0.054	0.468

**Notes**: Global Moran’s I was calculated using the contiguity-based spatial weight (queen contiguity), and the p-value was delivered by randomization with 999 permutations to test the significance of the actual data compared with the reference distribution for Moran’s I

**Table 2:** GWR results of community determinants of male suicide mortality in South Korea (2021)

	Distribution of GWR coefficients across 249 districts				Number of significant districts** (*N* = 249)	
	Q1 Coeff.	Median Coeff.	Q3 Coeff.	Global Coeff.*		
(Intercept)	-0.004	0.043	0.091	0.016		
**Aging index**	0.058	0.183	0.464	0.179	122	49.0%
**Single-person households**	0.225	0.294	0.346	0.278	217	87.1%
**Psychiatric clinics**	-0.201	-0.152	-0.014	-0.153	122	49.0%
**Unmet medical needs**	0.088	0.117	0.136	0.049	9	3.6%
AIC: 602.244 Residual sum of squares: 150.471			AICc: 629.764 Quasi-global R2: 0.373			

* Global coefficients correspond to values obtained using the ordinary least squares method, which does not consider spatial heterogeneity

** Number of districts where GWR coefficients were statistically significant among 249 districts in which their local t-values exceeded 1.96 or were below − 1.96

**Notes**: GWR, geographically weighted regression; AIC, Akaike information criterion; AICc, Akaike information criterion with correction. All variables are standardized. The GWR was performed based on a fixed bandwidth (= 76.921 *Km*) with a Gaussian Kernel function

### Electronic supplementary material

Below is the link to the electronic supplementary material.

Supplementary Material 1

## Data Availability

Data can be obtained from a third party and are not publicly available due to ethical concerns. Detailed information on the data sources is provided in the supplemental materials. Additional information can be obtained from the corresponding author upon reasonable request.
