# Supplementary material for: Spatially clustered patterns of suicide mortality rates in South Korea: a geographically weighted regression analysis
Source: BMC Public Health. 2024 Sep 2;24:2380. doi: 10.1186/s12889-024-19899-4 (PMC11367767; doi:10.1186/s12889-024-19899-4)
Supplement: Supplementary file 1 — Supplementary Material 1 [file 12889_2024_19899_MOESM1_ESM.docx]

**Supplemental materials**

**Spatially clustered patterns of suicide mortality rates in South Korea: A geographically weighted regression analysis**

**Appendix A:** Data sources and descriptive results of community determinants 2

**Appendix B**: Correlation between male suicide rates and suicide rate by age groups in South Korea (2021) 6

**Appendix C:** Geographically weighted regression model selection procedure 7

**Appendix D:** Distribution of the geographically weighted regression local R-squared values and information on the outlier 10

**Appendix E:** Sensitivity analysis of GWR with a total of 11 variables on male suicide mortality in South Korea (2021) 11

**Appendix F:** Geographical distribution of male suicide mortality based on administrative boundaries of South Korea (2021) 14

**Appendix G:** Local map of GWR coefficients for each community factor related to male suicide mortality in South Korea (2021) 15

**Appendix A: Data sources and descriptive results of community determinants**

South Korea has 17 cities and provinces, further subdivided into 250 districts. Since data were collected from 229 to 255 units, we adjusted those to 250 districts. For data from 229 districts, we applied representative values from the upper administrative division (district of ‘si’ unit) to the lower level (district of ‘gu’ unit). In contrast, data from the Community Health Survey, conducted across 255 public healthcare centers nationwide, required aggregation. We first calculated mean values for each of the 255 communities by accounting for the survey weights, strata, and clusters from the sample design. Subsequently, we averaged the community’s results to obtain representative values per district. Table S1 provides the data sources and variable definitions we used.

Table S2 presents the descriptive results of the community determinants in 250 districts of South Korea for 2021. The population over 65 years old was 2.36 times larger than that under 15 years old, and 34.15% of households were single-person households. Regarding economic environments, the gross regional domestic product (GRDP) per capita reached approximately 36,990 USD, but 2.60% of the economically active population was unemployed. Urbanization rates across districts averaged 76.96% and the road network index was 2.18, indicating a well-established traffic network and high degree of urbanization in South Korea. There were 2.65 psychiatric clinics and hospitals per 100,000 people. The 4.53% of the community residents experienced unmet medical utilization despite the need for medical care. The COVID-19 pandemic affected people’s daily lives such that 48.7% of people said that the crisis brought their daily living levels to a standstill by more than half. Regarding social relations, 69.34% of the residents in the community reported that they trusted and believed their neighbors. As a proxy for the cultural norm related to addressing mental distress, the indicator of usage in psychiatric counseling shows that only 20.15% of those suffering from enough distress to interfere with their daily lives had used psychiatric counseling services.

**Table S1.** Definition and source of data

| **Variable** | **Unit** | **Year** | **Region**  **Unit** | **Variable definition** | **Source (Publisher)** | | **Data**  **library** |
| --- | --- | --- | --- | --- | --- | --- | --- |
| **Deaths and study population** | | | | | | | |
| Suicide | Cases | 2021 | 250 | Deaths coded as intentional self-harm (X60–X84) | Cause of Death Statistics  (Statistics Korea) | | MDIS |
| Population size | No. of | 2021 | 250 | Number of populations based on resident registration in Korea | Population Statistics Based on Resident Registration (MOIS) | | KOSIS |
| Administrative boundaries | - | 2021 | 250 | Census administrative boundaries of Korea | Statistics Korea | | SGIS |
| **Demographic characteristics** | | | | | | | |
| Aging index | Index | 2021 | 250 | Ratio of the number of people aged 65 years and over to those aged 0 to 14 years (+65 years/<15 years) | Population Statistics Based on Resident Registration (MOIS) | | KOSIS |
| Single-person households | % | 2021 | 250 | Percentage of the number of single-person households out of total types of households | Population and Housing Census (Statistics Korea) | | KOSIS |
| **Economic characteristics** | | | | | | | |
| GRDP per capita | 1000 USD | 2021 | 229 | Gross Regional Domestic Product per capita (GRDP; Regional GDP) | Regional Income Statistics (Statistics Korea) | | KOSIS |
|  |  |  |  | Exchange rate in 2021: 1144.42 KW/USD | The bank of Korea | |  |
| Unemployment rate | % | 2021  2/2 | 229 | Percentage of the unemployed population among economically active population aged over 15 years old | Local Area Labour Force Survey, Economically Active Population Survey (Statistics Korea) | | KOSIS |
| **Neighborhood characteristics** | | | | | | | |
| Urbanization rate | % | 2021 | 229 | Percentage of people living in urban areas out of the total population in the region | Statistics of Urban Plan (LX) | | KOSIS |
| Road network index | Index | 2021 | 229 | Total length of roads $(Km$) per land area (${Km}^{2}$) and population (1000 people) in the area:  $\frac{Roads\left( Km \right)}{\sqrt{land area \left( {Km}^{2} \right)\times1000 people)}}$ | Roads | Statistics of Urban Plan (LX) | KOSIS |
|  |  |  |  |  | Population | Population Statistics Based on Resident Registration (MOIS) |  |
|  |  |  |  |  | Land area | Cadastral Statistics, (MOLIT) |  |
| Psychiatric clinics | No. per 100000 | 2021  4/4 | 250 | Number of psychiatric clinics and hospitals per 100,000 people | National Health Insurance Statistical Yearbook (NHIS,HIRA) | | KOSIS |
| Unmet medical needs | % | 2021 | 255 | Percentage of survey respondents who answered that they had needed medical care in the past year but had not received it | Community Health Survey  (KDCA) | | CHS website |
| **Environmental events** | | | | | | | |
| Disruption of daily life due to COVID-19 | % | 2021 | 255 | Percentage of survey respondents who answered that COVID-19 has halted daily life by more than 50% (compared with the period before the pandemic) | Community Health Survey (KDCA) | | KOSIS |
| **Social and cultural characteristics** | | | | | | | |
| Social trust | % | 2021 | 255 | Percentage of survey respondents who answered “yes” when they were asked if they believe and trust their neighbors | Community Health Survey (KDCA) | | KOSIS |
| Usage of psychiatric counseling services | % | 2021 | 255 | Percentage of survey respondents who have used professional counseling services among those who felt sadness or despair continuously for over two weeks or had suicidal thoughts last year | Community Health Survey (KDCA) | | CHS website |

**Abbreviation**: CHS, Community Health Survey; HIRA, Health Insurance Review and Assessment Service; KDCA, Korea Disease Control and Prevention Agency; KOSIS, KOrean Statistical Information Service; LX, Korea Land and Geospatial Informatix Corporation; MDIS, Micro Data Integrated System; MOIS, Ministry of the Interior and Safety; MOLIT, Ministry of Land, Infrastructure and Transport; NHIS, National Health Insurance Service; SGIS, Statistical Geographic Information Service.

**Data libraries:** CHS website (<https://chs.kdca.go.kr>); KOSIS (<https://kosis.kr>); MDIS (https://mdis.kostat.go.kr); SGIS (<http://sgis.kostat.go.kr)>.

**Table S2**. Descriptive results of the community environments in South Korea (2021)

| **Variables** | | **(unit)** | **Mean** | **SD** |
| --- | --- | --- | --- | --- |
| **Demographic characteristics** | | | | |
|  | Aging index | (index) | 2.36 | 1.55 |
|  | Single-person households | (%) | 34.15 | 5.10 |
| **Economic characteristics** | | | | |
|  | GRDP per capita | ($1000USD) | 36.99 | 34.33 |
|  | Unemployment rate | (%) | 2.60 | 1.47 |
| **Neighborhood characteristics** | | | | |
|  | Urbanization rate | (%) | 76.96 | 26.48 |
|  | Road network index | (Index) | 2.18 | 0.77 |
|  | Psychiatric clinics | (No. per 100,000) | 2.65 | 2.31 |
|  | Unmet medical needs | (%) | 4.53 | 2.14 |
| **Environmental events** | | | | |
|  | Disruption of daily life due to COVID-19 | (%) | 48.70 | 7.29 |
| **Social and cultural characteristics** | | | | |
|  | Social trust | (%) | 69.34 | 10.37 |
|  | Usage of psychiatric counseling services | (%) | 20.15 | 6.24 |

**Abbreviation:** SD, standard deviation; GRDP, gross regional domestic product.

**Appendix B: Correlation between male suicide rates and suicide rates by age groups in South Korea (2021)**

**
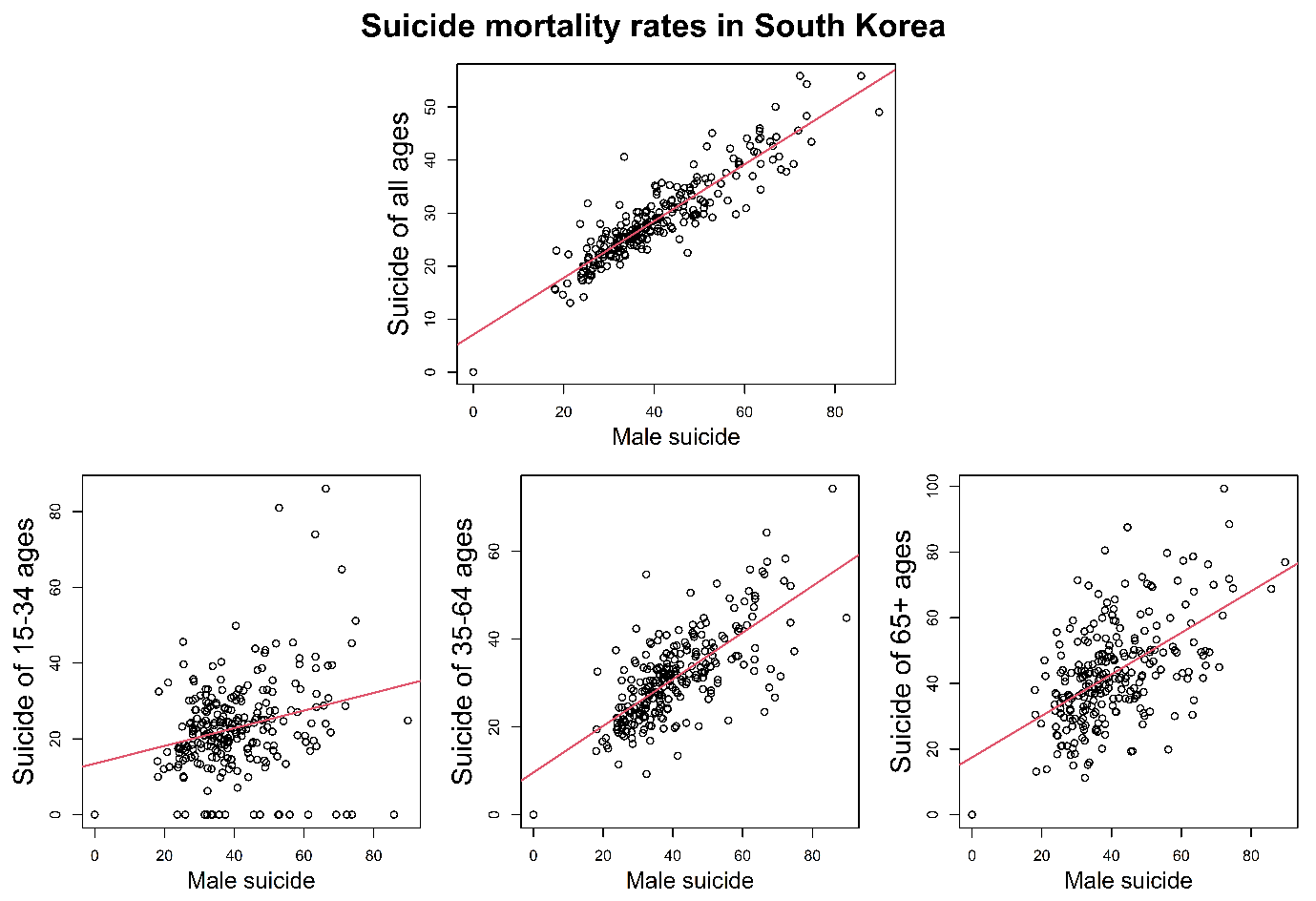
**

**Note:** Each circle indicates values from districts in South Korea (N=250).

**Figure S1**. Distribution of male suicide mortality rates and rates by age groups.

**Table S3.** Correlation between male suicide rates and suicide rate by age groups

| **(y, x)** | **Pearson correlation** | | **Univariate regression** |
| --- | --- | --- | --- |
|  | **Correlation Coefficient (*r*)** | ***p*-value** | **R-squared** |
| (Suicide of all ages, Male suicide) | 0.906 | <0.001 | 0.821 |
| (Suicide of 15-34, Male suicide) | 0.253 | <0.001 | 0.064 |
| (Suicide of 35-64, Male suicide) | 0.712 | <0.001 | 0.507 |
| (Suicide of 65+, Male suicide) | 0.548 | <0.001 | 0.301 |

**Appendix C: Geographically weighted regression model selection procedure**

To select an appropriate subset among the 11 variables, geographically weighted regression (GWR) model selection procedures were employed using an R function called *model.selection.gwr* [1]. Community determinants included the (1) aging index, (2) single-person household rate, (3) GRDP per capita, (4) unemployment rate, (5) urbanization rate, (6) road network index, (7) number of psychiatric clinics per 100,000 people, (8) unmet medical needs, (9) COVID-19 impact on daily life, (10) social trust, and (11) usage of psychiatric counseling services by patients were involved. In GWR model selection, we applied fixed bandwidth and Gaussian kernel options. As shown in Figure S2 and Table S4, the model including (1) aging index, (2) single-person household rate, (3) number of psychiatric clinics and hospitals, and (4) unmet medical needs reported the minimum Akaike information criterion with correction (AICc) value, indicating a relevant subset to explain male suicide mortality.

**
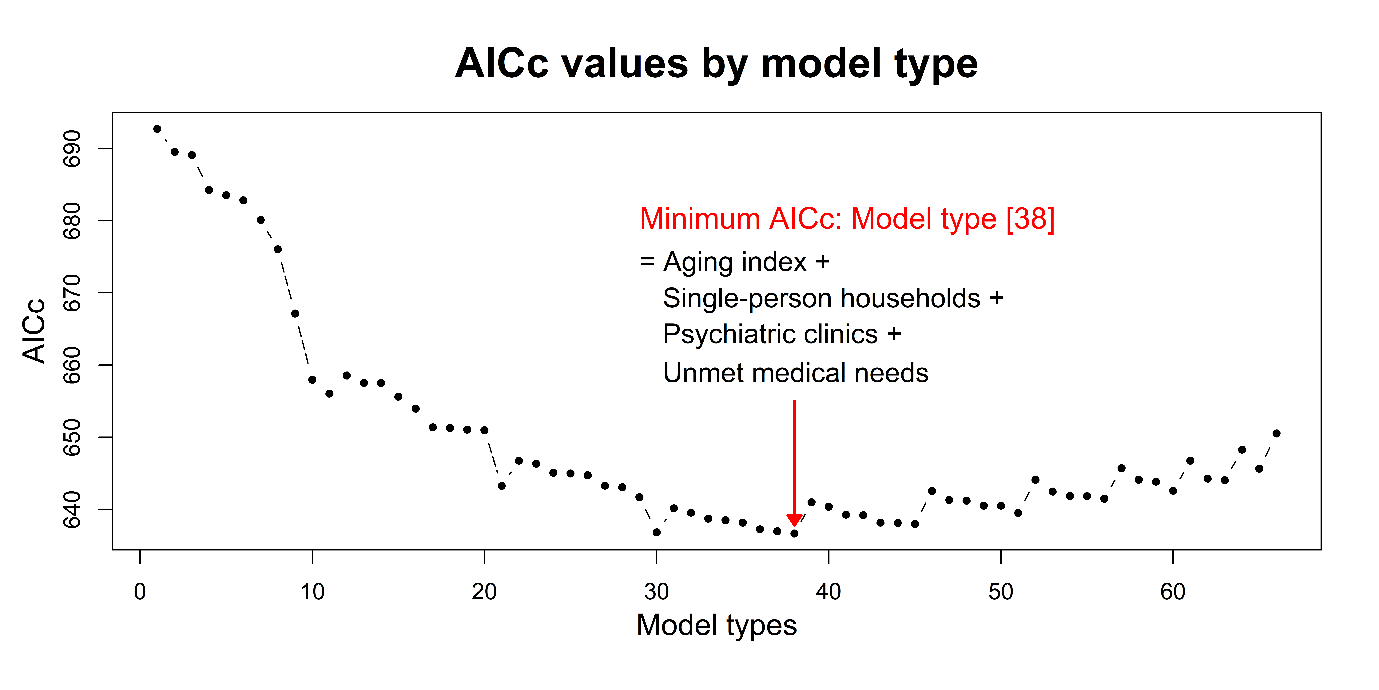
**

**Figure S2.** Akaike information criterion with correction (AICc) distributions by model types based on the geographically weighted regression model selection

**Table S4.** Model configuration based on geographically weighted regression model selection procedure

|  | **AICc** | **Model configuration** |
| --- | --- | --- |
| [1] | 692.7 | Suicide~GRDP |
| [2] | 689.5 | Suicide~Road |
| [3] | 689.1 | Suicide~Counseling |
| [4] | 684.2 | Suicide~COVID19 |
| [5] | 683.5 | Suicide~Unmet |
| [6] | 682.8 | Suicide~Clinics |
| [7] | 680.1 | Suicide~Trust |
| [8] | 676.0 | Suicide~Urban |
| [9] | 667.1 | Suicide~Unemploy |
| [10] | 657.9 | Suicide~Single |
| [11] | 656.0 | Suicide~Aging |
| [12] | 658.5 | Suicide~Aging+GRDP |
| [13] | 657.5 | Suicide~Aging+Urban |
| [14] | 657.5 | Suicide~Aging+Counseling |
| [15] | 655.6 | Suicide~Aging+Trust |
| [16] | 654.0 | Suicide~Aging+Road |
| [17] | 651.3 | Suicide~Aging+COVID19 |
| [18] | 651.3 | Suicide~Aging+Unemploy |
| [19] | 651.0 | Suicide~Aging+Clinics |
| [20] | 650.9 | Suicide~Aging+Unmet |
| [21] | 643.3 | Suicide~Aging+Single |
| [22] | 646.7 | Suicide~Aging+Single+GRDP |
| [23] | 646.3 | Suicide~Aging+Single+Urban |
| [24] | 645.1 | Suicide~Aging+Single+Road |
| [25] | 645.0 | Suicide~Aging+Single+Counseling |
| [26] | 644.7 | Suicide~Aging+Single+Trust |
| [27] | 643.3 | Suicide~Aging+Single+Unmet |
| [28] | 643.1 | Suicide~Aging+Single+COVID19 |
| [29] | 641.7 | Suicide~Aging+Single+Unemploy |
| [30] | 636.8 | Suicide~Aging+Single+Clinics |
| [31] | 640.1 | Suicide~Aging+Single+Clinics+Counseling |
| [32] | 639.5 | Suicide~Aging+Single+Clinics+Urban |
| [33] | 638.7 | Suicide~Aging+Single+Clinics+Trust |
| [34] | 638.5 | Suicide~Aging+Single+Clinics+Road |
| [35] | 638.2 | Suicide~Aging+Single+Clinics+GRDP |
| [36] | 637.2 | Suicide~Aging+Single+Clinics+Unemploy |
| [37] | 637.0 | Suicide~Aging+Single+Clinics+COVID19 |
| **[38]*** | **636.6** | **Suicide~Aging+Single+Clinics+Unmet** |
| [39] | 641.0 | Suicide~Aging+Single+Clinics+Unmet+Counseling |
| [40] | 640.4 | Suicide~Aging+Single+Clinics+Unmet+Urban |
| [41] | 639.3 | Suicide~Aging+Single+Clinics+Unmet+Road |
| [42] | 639.2 | Suicide~Aging+Single+Clinics+Unmet+Trust |
| [43] | 638.2 | Suicide~Aging+Single+Clinics+Unmet+Unemploy |
| [44] | 638.1 | Suicide~Aging+Single+Clinics+Unmet+COVID19 |
| [45] | 638.0 | Suicide~Aging+Single+Clinics+Unmet+GRDP |
| [46] | 642.6 | Suicide~Aging+Single+Clinics+Unmet+GRDP+Counseling |
| [47] | 641.3 | Suicide~Aging+Single+Clinics+Unmet+GRDP+Unemploy |
| [48] | 641.2 | Suicide~Aging+Single+Clinics+Unmet+GRDP+Trust |
| [49] | 640.5 | Suicide~Aging+Single+Clinics+Unmet+GRDP+Road |
| [50] | 640.5 | Suicide~Aging+Single+Clinics+Unmet+GRDP+Urban |
| [51] | 639.5 | Suicide~Aging+Single+Clinics+Unmet+GRDP+COVID19 |
| [52] | 644.1 | Suicide~Aging+Single+Clinics+Unmet+GRDP+COVID19+Counseling |
| [53] | 642.5 | Suicide~Aging+Single+Clinics+Unmet+GRDP+COVID19+Road |
| [54] | 641.8 | Suicide~Aging+Single+Clinics+Unmet+GRDP+COVID19+Urban |
| [55] | 641.8 | Suicide~Aging+Single+Clinics+Unmet+GRDP+COVID19+Unemploy |
| [56] | 641.5 | Suicide~Aging+Single+Clinics+Unmet+GRDP+COVID19+Trust |
| [57] | 645.7 | Suicide~Aging+Single+Clinics+Unmet+GRDP+COVID19+Trust+Counseling |
| [58] | 644.1 | Suicide~Aging+Single+Clinics+Unmet+GRDP+COVID19+Trust+Road |
| [59] | 643.8 | Suicide~Aging+Single+Clinics+Unmet+GRDP+COVID19+Trust+Unemploy |
| [60] | 642.6 | Suicide~Aging+Single+Clinics+Unmet+GRDP+COVID19+Trust+Urban |
| [61] | 646.7 | Suicide~Aging+Single+Clinics+Unmet+GRDP+COVID19+Trust+Urban+Counseling |
| [62] | 644.3 | Suicide~Aging+Single+Clinics+Unmet+GRDP+COVID19+Trust+Urban+Unemploy |
| [63] | 644.0 | Suicide~Aging+Single+Clinics+Unmet+GRDP+COVID19+Trust+Urban+Road |
| [64] | 648.3 | Suicide~Aging+Single+Clinics+Unmet+GRDP+COVID19+Trust+Urban+Road  +Counseling |
| [65] | 645.6 | Suicide~Aging+Single+Clinics+Unmet+GRDP+COVID19+Trust+Urban+Road  +Unemploy |
| [66] | 650.5 | Suicide~Aging+Single+Clinics+Unmet+GRDP+COVID19+Trust+Urban+Road  +Unemploy+Counseling |

***** The [numbers] correspond to the model types shown in Figure S2.

****** Final selected subset of independent variables

**Abbreviation**: AICc, Akaike information criterion with correction; Aging, Aging index; Single.H, Single-person household rates (%); GRDP, Gross regional domestic product per capita; Unemploy, Unemployment rates (%); Road, Road network index; Urban, Urbanization rates (%); Clinics, Number of psychiatric clinics and hospitals per 100000 people; Unmet, Unmet medical needs (%); COVID19, COVID-19 impact on daily life (%); Trust, Social trust (%); Counseling, Usage of psychiatric counseling rates by patients (%)

**Appendix D: Distribution of the geographically weighted regression local R-squared values and information on the outlier**

When fitting the GWR model to the 250 districts, one district showed a GWR local R-squared of 0.9; we regarded this as an outlier and excluded it. This is because if the value of the GWR local R-squared is particularly high, there can be multicollinearity between the local coefficients [2]. Therefore, we fitted 249 observations to the GWR model in the main text of the manuscript.


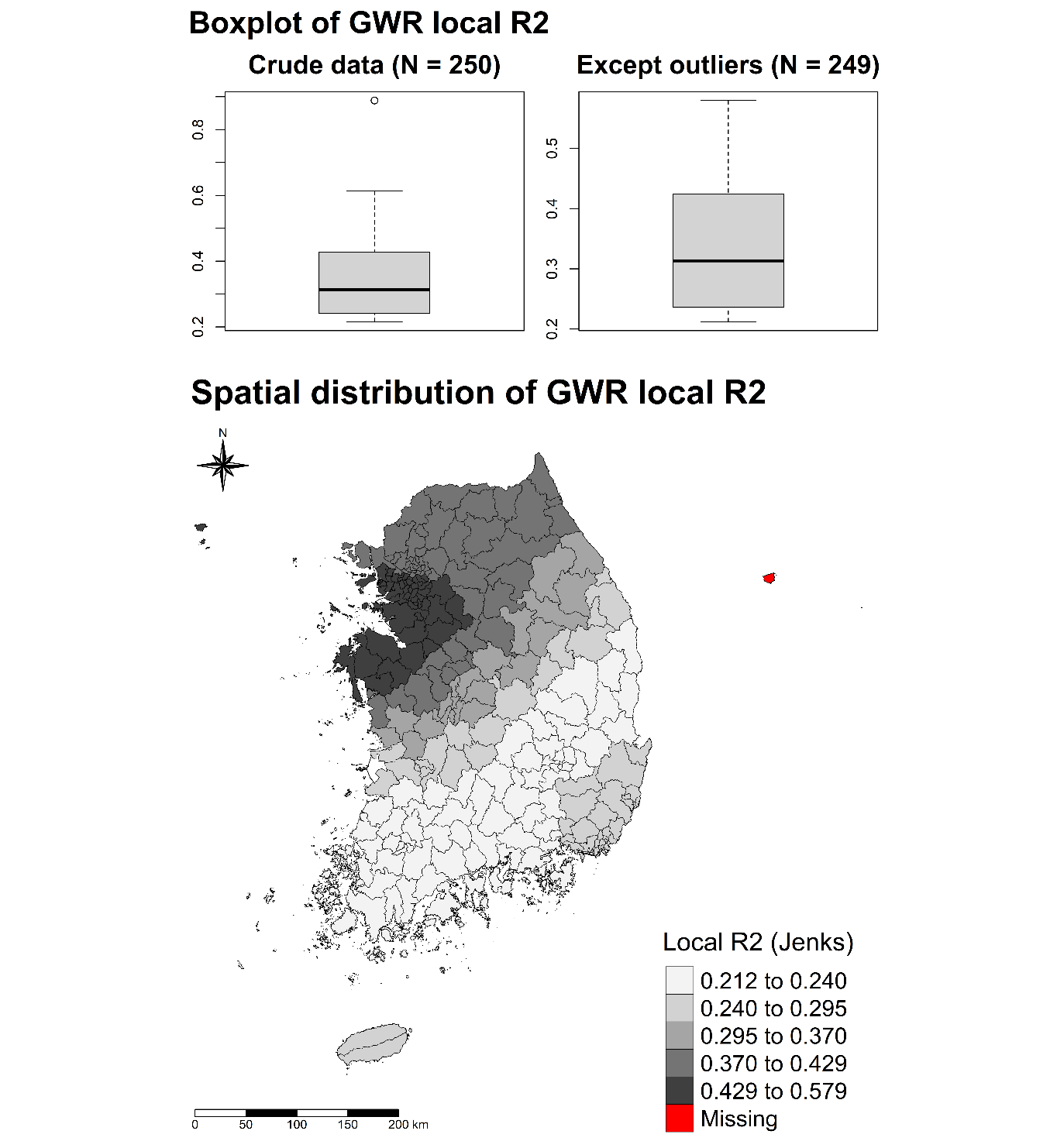


**Note**: Red marked as missing in the figure corresponds to the outlier that we excluded.

**Figure S3**. Geographical distribution of GWR local R-squared values on male suicide in South Korea

**Appendix E. Sensitivity analysis of GWR with a total of 11 variables on male suicide mortality in South Korea (2021)**

To validate our selected subset of variables, we performed a sensitivity analysis with four types of additional analyses. These compare the GWR to ordinary least squares regression (OLS) and models with 4 selected variables to a total set of 11 variables.

Table S5 shows that each GWR model has a lower residual sum of squares and a higher level of R-squared compared to OLS. The global test for GWR [3] concludes that there is a spatial effect, and the GWR model is more appropriate than the OLS model in terms of goodness of fit. The AICc value is the lowest in the GWR model with 4 variables across the 4 types of models.

Table S6 presents each variable’s coefficients and their statistical significance. After GWR model selection, the range of GWR coefficients for the 4 variables did not differ much from the results of the 11-variable model, and the VIF of each variable in OLS decreased. These considerations advocate for the necessity of the GWR variable selection process.

**Table S5.** Model comparison between models with 11 variables and 4 variables for male suicide mortality using OLS and GWR (2021)

|  | **Model with 11 variables** | | **Model with 4 variables** | |
| --- | --- | --- | --- | --- |
|  | **GWR** | **OLS** | **GWR** | **OLS** |
| N* | 249 | 249 | 249 | 249 |
| Bandwidth(fixed)** | 105.6 km | . | 76.9 km | . |
| AICc | 643.1 | 649.3 | 629.8 | 647.9 |
| R-squared*** | 0.410 | 0.227 | 0.373 | 0.207 |
| Residual sum of squares (RSS) | 141.5 | 177.1 | 150.5 | 187.2 |
| **Global test for GWR improvement** |  | |  | |
| RSS improvement of GWR | 35.6 | | 36.7 | |
| Spatial non-stationarity test: F(2) test | p-value = 0.013 | | p-value = 0.001 | |
| Goodness of fit test: ANOVA test | p-value = 0.049 | | p-value = 0.049 | |

***** One district was excluded from the 250 districts due to its extremely high local R-squared value, which implies overfitting.

****** A Gaussian kernel function with fixed bandwidth was applied in GWR.

******* Quasi-global R2 for the GWR model; Adjusted R-squared for OLS.

**Note**: F(2) test refers to Leung et al. (2000)'s study, while the ANOVA test is from Brunsdon, Fotheringham, and Charlton (2002).

**Table S6.** Coefficients from OLS and GWR models with 11 variables and 4 variables for male suicide mortality (2021)

|  | **GWR**  **with 11 variables** | | | |  | **OLS**  **with 11 variables** | | |  | **GWR**  **with 4 variables** | | | |  | **OLS**  **with 4 variables** | | |
| --- | --- | --- | --- | --- | --- | --- | --- | --- | --- | --- | --- | --- | --- | --- | --- | --- | --- |
|  | **Q1**  **Coeff.** | **Q2**  **Coeff.** | **Q3**  **Coeff.** | **#Sig.** |  | **Coeff.** | **Pr(>\|t\|)** | **VIF** |  | **Q1**  **Coeff.** | **Q2**  **Coeff.** | **Q3**  **Coeff.** | **#Sig.** |  | **Coeff.** | **Pr(>\|t\|)** | **VIF** |
| Aging index | 0.04 | 0.12 | 0.32 | 82 |  | 0.17 | 0.13 | 3.91 |  | 0.06 | 0.18 | 0.46 | 122 |  | 0.18 | 0.02* | 1.81 |
| Single-person households | 0.27 | 0.30 | 0.33 | 235 |  | 0.28 | 0.00*** | 1.87 |  | 0.22 | 0.29 | 0.35 | 217 |  | 0.28 | 0.00*** | 1.72 |
| Psychiatric clinics | -0.23 | -0.11 | -0.01 | 115 |  | -0.14 | 0.04* | 1.40 |  | -0.20 | -0.15 | -0.01 | 122 |  | -0.15 | 0.01** | 1.11 |
| Unmet medical needs | 0.08 | 0.09 | 0.10 | 0 |  | 0.06 | 0.36 | 1.16 |  | 0.09 | 0.12 | 0.14 | 9 |  | 0.05 | 0.40 | 1.05 |
| GRDP per capita | 0.06 | 0.08 | 0.10 | 8 |  | 0.06 | 0.34 | 1.17 |  | . | . | . | . |  | . | . | . |
| Unemployment rate | -0.23 | -0.21 | -0.20 | 72 |  | -0.24 | 0.01* | 2.83 |  | . | . | . | . |  | . | . | . |
| Urbanization rate | 0.07 | 0.15 | 0.23 | 15 |  | 0.23 | 0.05† | 4.55 |  | . | . | . | . |  | . | . | . |
| Road network index | -0.11 | -0.05 | 0.02 | 40 |  | -0.04 | 0.47 | 1.06 |  | . | . | . | . |  | . | . | . |
| Disruption of daily life due to COVID-19 | -0.03 | 0.04 | 0.07 | 0 |  | -0.01 | 0.90 | 1.25 |  | . | . | . | . |  | . | . | . |
| Social trust | -0.06 | 0.05 | 0.14 | 16 |  | 0.07 | 0.42 | 2.32 |  | . | . | . | . |  | . | . | . |
| Usage of psychiatric counseling services | -0.09 | -0.07 | -0.07 | 0 |  | -0.07 | 0.24 | 1.21 |  | . | . | . | . |  | . | . | . |
| (Intercept) | 0.04 | 0.05 | 0.05 | . |  | 0.02 | 0.75 | . |  | 0.00 | 0.04 | 0.09 | . |  | 0.02 | 0.77 | . |

**Significance**: *** 0.001; ** 0.01; * 0.05 ; † 0.1

**Abbreviation**: Coeff., Coefficient; #Sig., The number of significant regions

**Appendix F: Geographical distribution of male suicide mortality based on administrative boundaries of South Korea (2021)**

**
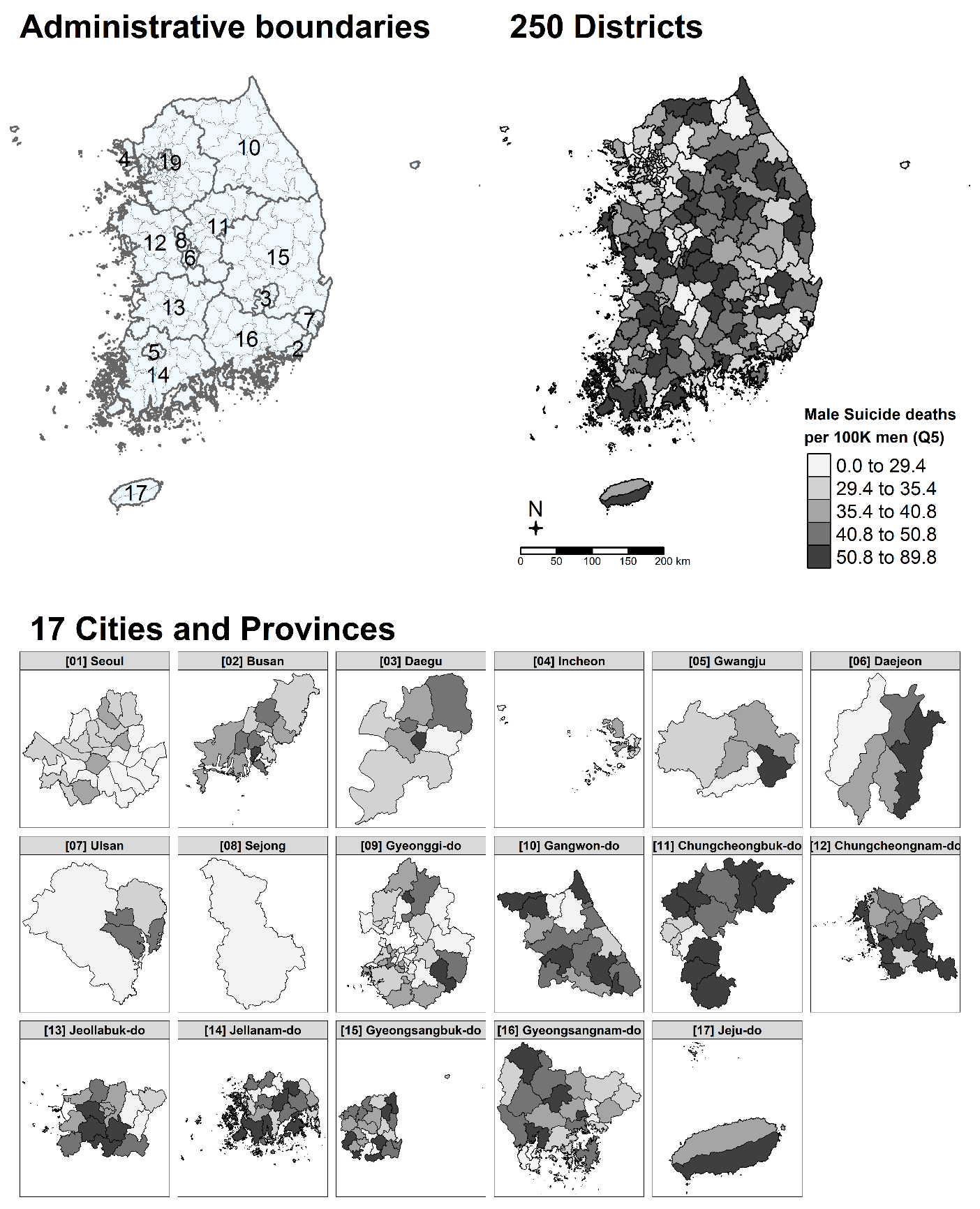
**

**Note:** Figure S4 is divided into quintiles across 250 districts, illustrating bundling by city and provincial boundaries.

**Figure S4.** (A) Administrative boundaries of South Korea and male suicide mortality rates (B) by district and (C) by city and province levels

**Appendix G: Local map of GWR coefficients for each community factor related to male suicide mortality in South Korea (2021)**

**
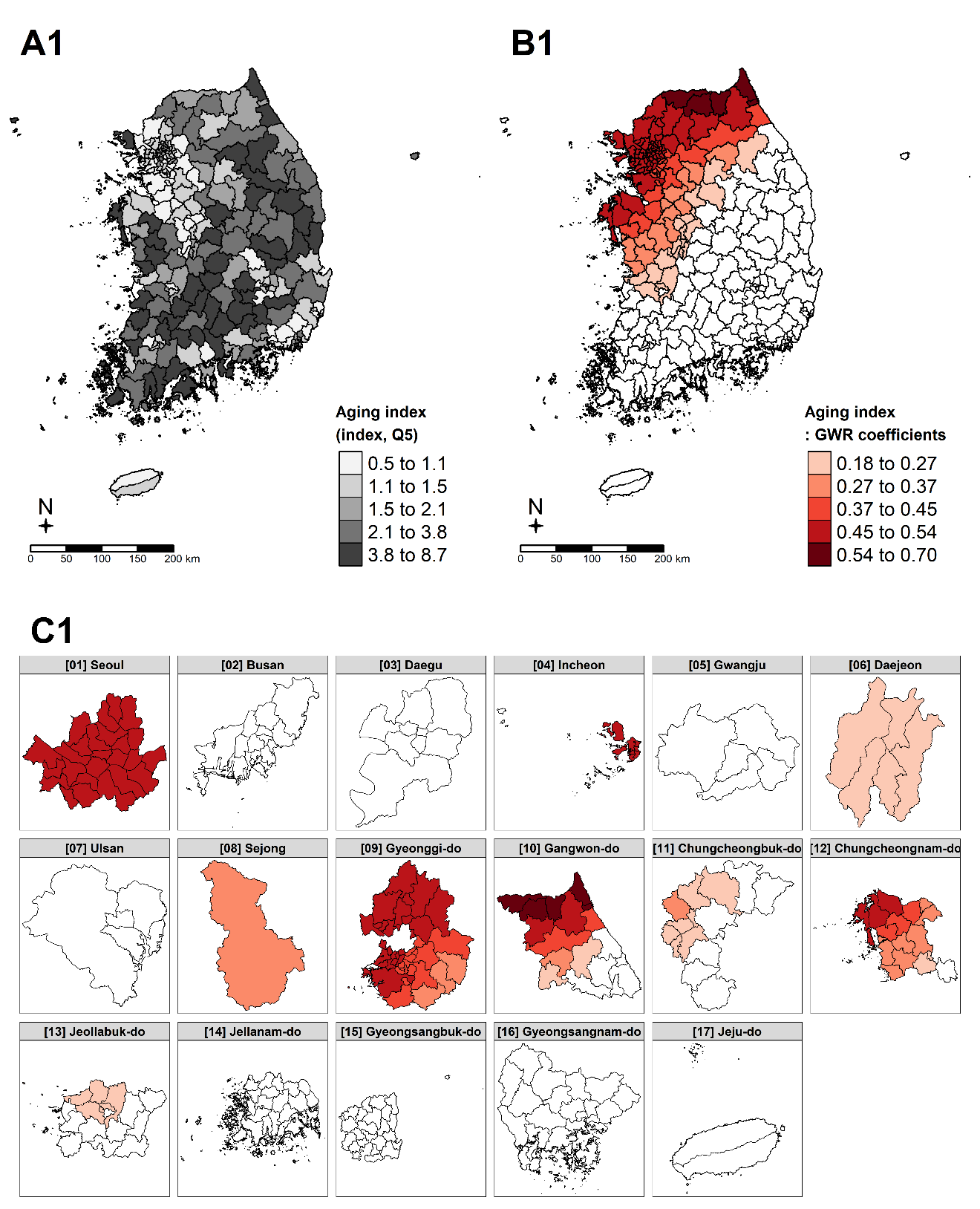
**

**Note:** Figure A is divided into quintiles, while B and C are colored based on Jenks (natural breaks) classes.

**Figure S5.** Geographical distribution of (A1) aging index and (B1 their GWR coefficients for male suicide mortality across 250 districts, with (C1) depicted by city and province levels for the coefficients

**
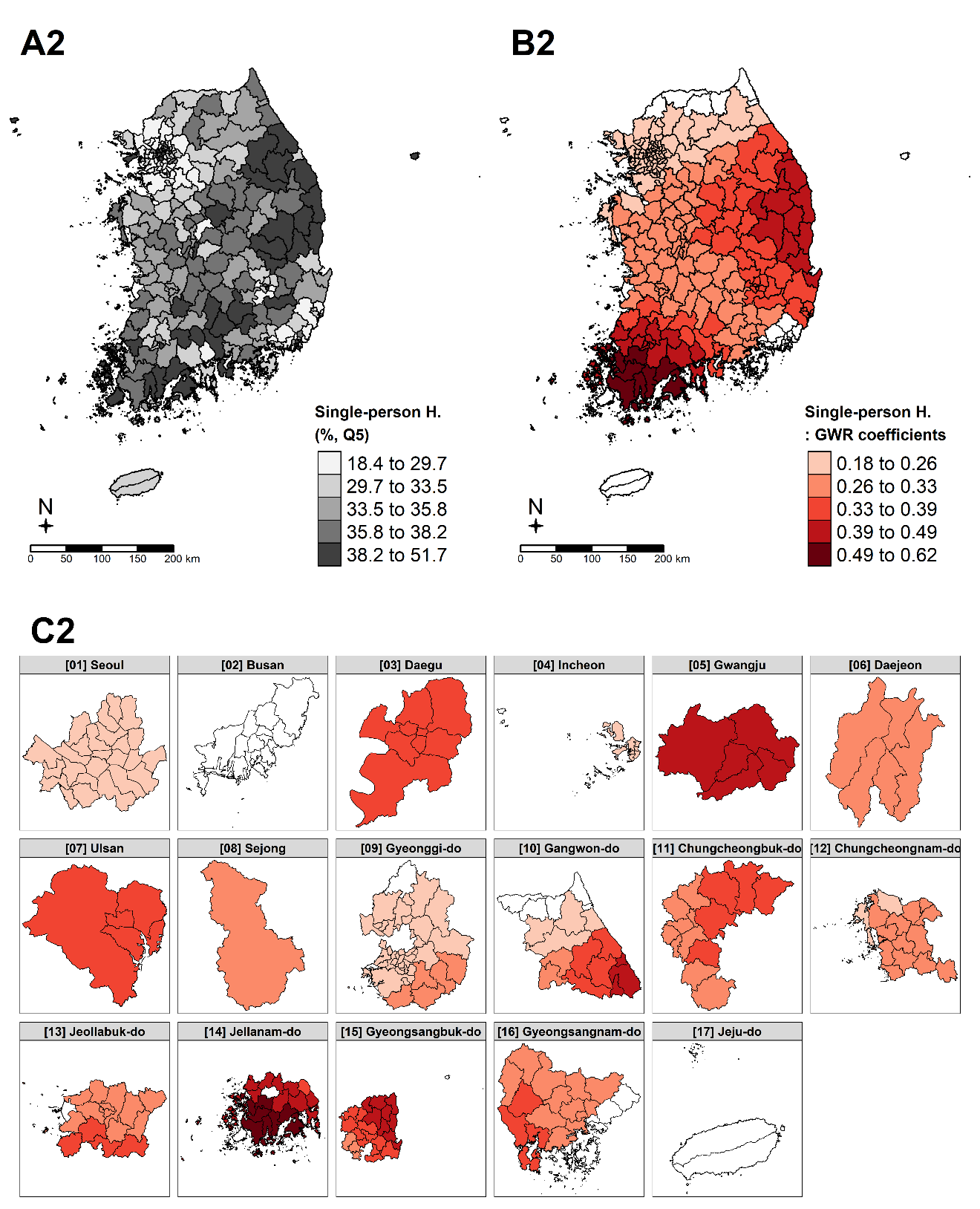
**

**Note:** Figure A is divided into quintiles, while B and C are colored based on Jenks (natural breaks) classes.

**Figure S6.** Geographical distribution of (A2) the single-person household rates and (B2) their GWR coefficients for male suicide mortality across 250 districts, with (C2) depicted by city and province levels for the coefficients

**
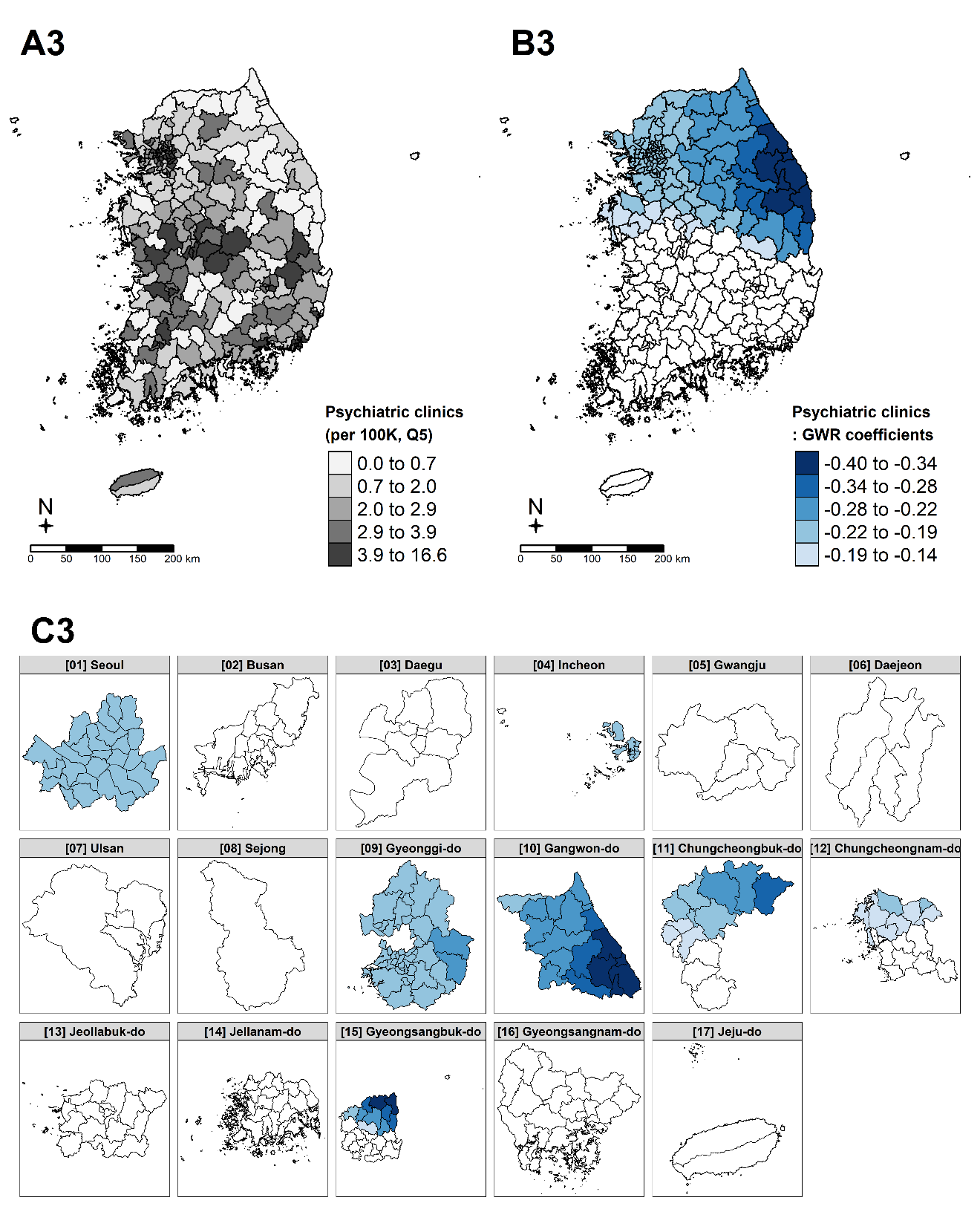
**

**Note:** Figure A is divided into quintiles, while B and C are colored based on Jenks (natural breaks) classes.

**Figure S7.** Geographical distribution of (A3) the number of psychiatric clinics and (B3) their GWR coefficients for male suicide mortality across 250 districts, with (C3) depicted by city and province levels for the coefficients

**
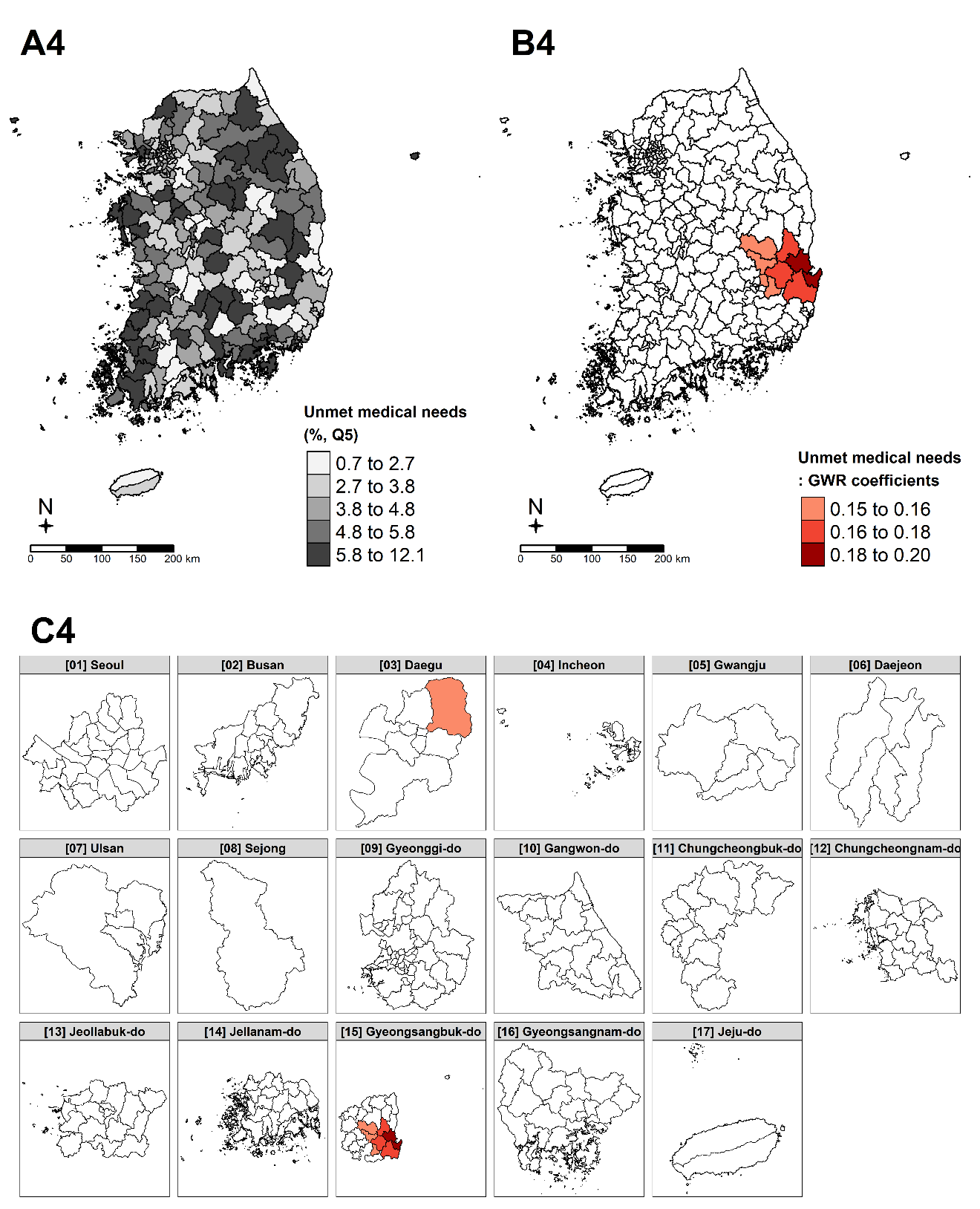
**

**Note:** Figure A is divided into quintiles, while B and C are colored based on Jenks (natural breaks) classes.

**Figure S8.** Geographical distribution of (A4) unmet medical needs and (B4) their GWR coefficients for male suicide mortality across 250 districts, with (C4) depicted by city and province levels for the coefficients

**Reference**

1. Gollini I, Lu B, Charlton M, Brunsdon C, Harris P: **GWmodel: An R Package for Exploring Spatial Heterogeneity Using Geographically Weighted Models**. *Journal of Statistical Software* 2015, **63**(17):1 - 50.

2. Wheeler D, Tiefelsdorf M: **Multicollinearity and correlation among local regression coefficients in geographically weighted regression**. *Journal of Geographical Systems* 2005, **7**(2):161-187.

3. Leung Y, Mei C-L, Zhang W-X: **Statistical Tests for Spatial Nonstationarity Based on the Geographically Weighted Regression Model**. *Environment and Planning A: Economy and Space* 2000, **32**(1):9-32.
